# Development of Water‐Dispersible CuS Nanoparticles for Chemo–Photothermal Therapy and Photoacoustic Application

**DOI:** 10.1002/cphc.202500016

**Published:** 2025-07-03

**Authors:** Sonali Gupta, Bijaideep Dutta, Subhadip Paul, Ratan K. Saha, Kanhu C. Barick, Puthusserickal A. Hassan

**Affiliations:** ^1^ Chemistry Division Bhabha Atomic Research Centre Trombay Mumbai 400085 India; ^2^ Homi Bhabha National Institute Anushaktinagar Mumbai 400094 India; ^3^ Department of Applied Sciences Indian Institute of Information Technology Allahabad Allahabad 211012 India

**Keywords:** chemotherapies, CuS nanoparticles, doxorubicin, photoacoustic, photothermal

## Abstract

Photothermal therapy (PTT) is a promising treatment option for cancer. However, biocompatibility, water dispersibility, and good photoresponsiveness are necessary for photothermal agents to be used in PTT. Herein, a soft chemical approach is reported for the design and development of phosphorylethanolamine‐functionalized CuS nanoparticles (PEA‐CuS NPs) using phosphorylethanolamine (PEA) as a surface passivation agent for combinatorial chemo–photothermal therapy. X‐ray diffraction and transmission electron microscopy analyses proved the successful formation of the green covellite CuS phase with an average particle size of 13 nm, while Fourier transform infrared spectroscopy and light scattering measurements demonstrated their surface functionalization with the PEA ligand. The PEA‐CuS NPs have exhibited good photothermal heating efficacy upon irradiation of 980 nm laser light. Moreover, the in vitro studies have shown substantial cellular uptake of PEA‐CuS NPs in MCF‐7 cell lines and their dose‐dependent cytotoxicity (83.3 and 65.4% cell‐killing efficacy by 100 and 50 μg mL^−1^ of PEA‐CuS NPs, respectively) upon 980 nm laser irradiation (0.65 W power). The developed PEA‐CuS NPs have also demonstrated very high acoustic signal under 690 and 530 nm laser light irradiation. Thus, these water‐dispersible PEA‐CuS NPs can be served as a promising candidate for image‐guided combinatorial therapy involving chemotherapy and PTT.

## Graphical abstract

Water‐dispersible CuS nanoparticles are developed by the soft chemical approach using phosphorylethanolamine as a surface passivation agent for chemo–photothermal therapy and photoacoustic application.

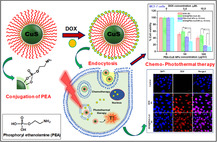

## 1 Introduction

In terms of death rate, cancer is the second most frequent disease even though there are many therapeutic modalities accessible for its treatment.^[^ ^1^, ^2^ ^]^ The main obstacles to obtain the best possible treatment results from the conventional chemotherapy and traditional medications include poor tumor‐targeting ability, undesired side effects, and multidrug resistance.^[^ ^3^, ^4^, ^5^, ^6^ ^]^ Because of its high tissue transparency, noninvasive manipulation, low normal cell toxicity, and reduced patient compliance, photoinduced therapy—photothermal therapy (PTT) and photodynamic therapy (PDT)—is becoming more and more popular.^[^ ^7^, ^8^, ^9^, ^10^ ^]^ PTT employs photothermal agents (PTAs), which generate heat and boost the temperature of the tumor microenvironment to 42–43 °C when exposed to near‐infrared (NIR) light, thereby killing the cancer cells.^[^ ^11^, ^12^, ^13^, ^14^, ^15^, ^16^, ^17^ ^]^ On the other hand, in PDT, photosensitizers are used to generate reactive oxygen species (ROS) after being irradiated with a photon with a particular wavelength, and subsequently damage the cancer cells, acting as a therapeutic module.^[^ ^18^, ^19^, ^20^ ^]^ New concepts for cancer treatment have recently been made possible with the combination of chemotherapy and photothermal therapeutic modality. It has been shown that PTT combined with chemotherapy improves the therapeutic efficacy in comparison to their individual counterparts.^[^ ^21^, ^22^, ^23^, ^24^ ^]^ To date, photothermal therapeutic systems have been created using a range of PTAs, including noble metal nanostructures, carbon‐based materials, and semiconductor compounds. Among them, Au‐ and Pd‐based nanostructures are expensive with poor photostability, and follow a complicated synthetic process, whereas carbon‐based nanomaterials possess low photothermal conversion efficiency.^[^ ^25^, ^26^ ^]^ The usage of semiconductor nanoparticles are also limited due to a certain degree of toxicity with the presence of toxic elements, like Cd, Hg, Pb, etc.^[^ ^27^, ^28^ ^]^

These issues have attracted the interest of researchers in developing copper chalcogenides (Cu_*x*_S, Cu_*x*_Se, and Cu_*x*_Te, where *x* = 1,2)‐based PTAs, which exhibit plasmonic properties typically found in metal nanoparticles (such as Au and Ag), along with excitonic properties common to semiconductor nanoparticles.^[^ ^29^, ^30^, ^31^, ^32^ ^]^ Unlike metals with free electrons, plasmonic behavior in copper chalcogenides arises from the collective oscillation of holes, a phenomenon triggered by oxidation that leads to the creation of Cu vacancies.^[^ ^33^, ^34^ ^]^ In contrast to other metal‐semiconductor heterostructures, this integration of electrical and photonic properties in a single nanoscale entity opens up new possibilities for applications in quantum information processing, nonlinear optics, and light harvesting.^[^ ^35^ ^]^ Moreover, Cu_*x*_S_*y*_ structures with different compositions have been extensively used for both in vitro and in vivo biomedical applications.^[^ ^36^, ^37^ ^]^ This has resulted in the development of various preparation methodologies such as chemical precipitation, high temperature thermal decomposition, solvothermal synthesis, hot injection, and microwave‐based processes for the preparation of Cu_*x*_S_*y*_ NPs.^[^ ^30^, ^38^, ^39^, ^40^, ^41^ ^]^ Among these, the chemical precipitation‐based soft chemical approach offers a simple and cost‐effective route for synthesizing covellite CuS NPs. However, there are significant challenges that still lie in the preparation of water‐dispersible CuS NPs of controlled shape and size with suitable surface functionality for chemo–photothermal therapy applications. Therefore, the development of surface‐functionalized colloidally stable CuS NPs is worthwhile.

In this aspect, we report the development of water‐dispersible phosphorylethanolamine‐functionalized CuS NPs (PEA‐CuS NPs), using a soft chemical approach that not only provides long‐term colloidal stability of particles but also generates a particular site for binding of the anticancer drug, doxorubicin hydrochloride (DOX). The developed PEA‐CuS NPs exhibited strong NIR absorption and good photothermal heating efficacy under the irradiation of NIR light (980 nm). These PEA‐CuS NPs, in associating with DOX, showed enhanced toxicity toward breast cancer cells (MCF‐7) under NIR light, indicating good chemo–photothermal efficacy. Moreover, these PEA‐CuS NPs showed an admirable ability to generate an acoustic signal when exposed to laser light over a varied concentration range. Specifically, the excellent photostability, good photoacoustic (PA) signal‐generating ability, and integrated chemo‐photothermal efficacy make them potential agents for cancer therapy.

## 2 Experimental Section

### 2.1 Materials

Cu (II) chloride dihydrate (CuCl_2_.2H_2_O), sodium sulfide (Na_2_S), sodium hydroxide (NaOH), and dimethyl sulfoxide (DMSO) were purchased from SDFCL, India. *O*‐phosphorylethanolamine (PEA) was purchased from TIC Pvt. Ltd., India, Dulbecco's modified eagle medium (DMEM), fetal bovine serum (FBS), antibiotics, MTT reagent, DAPI dihydrochloride, and dialysis membrane‐60 were procured from HiMedia Laboratories Pvt. Ltd., India. DOX and MCF‐7 cells were received as kind gifts from the Advanced Centre for Treatment, Research and Education in Cancer (ACTREC), Navi Mumbai and Radiation Biology & Health Science Division (RB&HSD), BARC, Mumbai, respectively.

All the aqueous solutions were prepared using deionized water (DI) from a Millipore Milli Q system (resistivity ≈18 MΩ cm). The acetate buffer (AB, pH 5.0), phosphate buffer (PB, pH 6.5), and phosphate buffered saline (PBS, pH 7.4) were prepared using standard protocols. All the chemicals used were of AR grade unless otherwise specified, and used as such without further treatment.

### 2.2 Synthesis of PEA‐CuS NPs

**Scheme 1:**
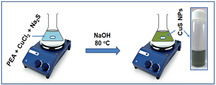
Pictorial representation of the synthesis protocol of the PEA‐CuS NPs.

Highly water‐dispersible, PEA‐CuS NPs were synthesized using a soft chemical approach, with PEA serving as the surface passivating agent. Aqueous colloidal‐stabilized CuS NPs were achieved at a molar ratio of Cu^+2^:S^2−^:PEA of 1:3:3. In a typical synthesis, 0.169 g of PEA was dissolved in 100 mL of Milli Q water with stirring for 15 min at 80 °C. Subsequently, 2 mL of 0.2 M CuCl_2_ (0.4 mmol) was added to the above solution. After 20 min, a 0.1 M NaOH solution was added dropwise to maintain a basic pH of 8–9, and the reaction was continued for another 10 min with stirring at the same temperature. Then, 6 mL of 0.2 M Na_2_S (1.2 mmol) was introduced, and the reaction solution was stirred for an additional period of 60 min at 80 °C. The resulting dark‐green PEA‐CuS NPs solution was dialyzed against the Milli Q water for 24 h to remove unreacted chemicals and by‐products. A portion of this solution was dried at 50°C in an oven for further characterization. Solution‐phase analyses were performed directly on the dialyzed PEA‐CuS NPs suspension. The schematic representation of the synthesis of the PEA‐CuS NPs is shown in **Scheme** **1**.

### 2.3 Characterizations

The structural/microstructural studies and surface functionalization were performed by X‐ray diffraction (XRD, Rigaku X‐ray diffractometer), transmission electron microscopy (TEM, Themis 300 G3, Thermo Scientific) and Fourier transform infrared spectroscopy (FTIR, Bomen MB series). The average hydrodynamic diameter (H_D_) and zeta potential (ZP) measurements were performed using Litesizer (Anton Paar). The absorption spectra were recorded using an UV–vis–NIR spectrophotometer (JASCO V‐670 UV–vis–NIR spectrophotometer). The concentration of Cu in the aqueous suspension and powder form of the PEA‐CuS NPs were measured using atomic absorption spectrometer (AAS, Analytik Jena).

### 2.4 Photothermal Heating Efficacy and Drug Loading/Release Studies

Photothermal heating studies were conducted by irradiating a 1 mL aqueous suspension of the synthesized PEA‐CuS NPs with NIR light (980 nm). The concentrations of the PEA‐CuS NPs, expressed as Cu, were 50, 100, and 150 μg mL^−1^, and the suspensions were exposed to varying laser powers ranging from 0.45 to 0.85 W. The temperature change in the solution was recorded every 10 sec using an optical fiber‐based temperature sensor, with an accuracy of ± 0.1 °C. To assess photostability, an aqueous suspension of the PEA‐CuS NPs (50 μg mL^−1^ Cu, 0.65 W) was subjected to 6 cycles of irradiation with a 980 nm NIR laser (heating–cooling curve). The temperature rise was also measured with a high‐resolution infrared (IR) camera (Thermal Imager Testo 875‐1) and analyzed by a thermography software (Testo IR Soft Software, version 3.1).

For the dual therapy approach, the anticancer drug, DOX was loaded onto the surface of the PEA‐CuS NPs. The loading procedure involved mixing 0.5 mL of an aqueous DOX solution (1 mg mL^−1^) with 5 mg of the PEA‐CuS NPs suspended in 2.5 mL of DI water. This mixture was vortex in the dark for 1 h (drug to particle ratio 1:10). After the completion of vortexing, the DOX‐loaded PEA‐CuS NPs (DOX@PEA‐CuS NPs) were separated by centrifugation at 5000 rpm for 5 min and subsequently washed with DI water. The absorbance spectra of the supernatant collected after the separation of the DOX@PEA‐CuS NPs along with the pure DOX solution (of the same concentration) were recorded using a JASCO V650 UV–vis spectrophotometer. The absorbance intensity of the supernatant at its maximum wavelength, including the washed drug molecule, was compared to that of the pure DOX solution to determine the loading efficiency. The loading efficiency was calculated by using the following relation:

$$\text{Loading efficiency}(\%)=\frac{A_{\text{DOX}}-A_{S}-A_{W}}{A_{\text{DOX}}}\times 100$$

where *A*_DOX_ is the absorbance of pure DOX; *A*_S_ is the absorbance of supernatant; and *A*_W_ is the absorbance of the washed DOX which is physically adsorbed. Further, the drug loading content was determined as follows:

$$\text{Drug}\;\text{loading content}(\%)=\frac{\text{weight of encapsulated}\text{ DOX}}{\text{weight of nanocariers}}\times 100$$

The pH‐dependent drug release study was conducted under reservoir–sink conditions (pH 5 vs pH 5, pH 6.5 vs pH 6.5, and pH 7.4 vs pH 7.4). For this study, 5 mg of the DOX@PEA‐CuS NPs was immersed in 3 mL of buffer solution of pH 5/pH6.5/pH 7.4, and then added into a dialysis bag. The dialysis was carried out against 100 mL of the respective sink medium under constant stirring at 37 °C, to stimulate the cellular environment. At predetermined time intervals, 1 mL of the external medium was removed and replaced with fresh medium to maintain the sink conditions. The amount of DOX released was quantified by measuring the fluorescence emission of the collected sample at 585 nm with an excitation wavelength of 490 nm, using a microplate reader (Gen 1.0.5, SYNERGY/H1 micro plate reader; BioTeK, Germany). Each experiment was performed three times, and the respective standard deviation (SD) was reported in the plots.

### 2.5 In Vitro Cytotoxicity and Photothermal Performance by MTT Assay

In vitro studies of PEA‐CuS NPs, DOX@PEA‐CuS NPs, and pure DOX were observed in the presence and absence of NIR irradiation on the MCF‐7 cell line using the MTT assay. 1 × 10^4^ cells per well were seeded in 96 well plates in complete culture medium at 37 °C and 5% CO_2_, overnight. After overnight incubation, the cells were treated with synthesized systems by replenishing fresh media containing different concentration of respective systems, and incubated for 24 h at 37 °C and 5% CO_2_. The MTT assay was performed and the relative cell viabilities were determined as reported earlier.^[^ ^23^, ^24^ ^]^ In brief, from each well the culture medium was removed and supplied with fresh medium containing 0.5 mg mL^−1^ of MTT and further incubated for 3‐4 h. After incubation, the MTT solution was removed and 100 μL of DMSO was added to each well for solubilizing the formed formazan crystals. The absorbance of each well was measured using a microplate reader at 570 nm to determine the cytotoxicity of the formulations. The % cell viability was obtained by comparing it with the viability of the control cells, which was considered as 100%. Each experiment was performed in triplicate, and the standard deviation was mentioned in the plot.

The in vitro photothermal toxicity of the formulated NPs in the presence and absence of the DOX was also determined by the MTT assay under irradiation of 980 nm laser light. For this, cells were treated with PEA‐CuS NPs and DOX@PEA‐CuS NPs for 18 h at 37 °C and 5% CO_2_. After 18 h, in order to remove excess particles, the culture medium was removed and the cells were washed with PBS. After washing, the cells were supplied with fresh media and irradiated for 5 min and 10 min under 980 nm laser at a power of 0.65 W. Then, the NIR light‐treated cells were further incubated overnight at 37 °C and 5% CO_2_. After the incubation period, the MTT assay was performed, and the relative cell viabilities were determined as discussed above. The morphological changes of the cells after treatment at different time intervals (after incubation) were captured using a brightfield microscope.

### 2.6 Cellular Internalization Studies by Confocal Microscopy

Cellular internalization studies were conducted on the MCF‐7 cells using confocal laser scanning microscopy (CLSM). For CLSM imaging, cells (0.5 × 10^6^) were seeded onto glass cover slips and cultured overnight. Subsequently, the cells were treated with the DOX and DOX@PEA‐CuS NPs at a DOX concentration of 4 μM for 3 and 6 h under standard culture conditions. Following treatment, the cells were washed with the PBS, fixed with 4% paraformaldehyde, and permeabilized using 0.1% triton X‐100. The cells were then mounted on a glass slide using a cell mounting medium (Invitrogen, USA) containing DAPI for nuclear staining. Imaging was performed using confocal microscopy (FV 3000, Olympus) with a red laser for the DOX and a blue laser for the DAPI.

### 2.7 PA Spectroscopic Study

**Scheme 2:**
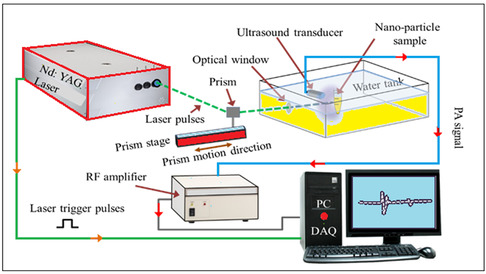
Schematic diagram of the setup used for the PA experiments.

The schematic diagram of the PA experimental setup is shown in **Scheme** **2**. It has a tunable Nd: YAG laser system (EKSPLA, NT300 series) with pulses of 6 ns width and 10 Hz pulse repetition frequency (PRF). This equipment has the capability to provide a wide range of wavelengths (335–2500 nm), with the exclusion of the optical range from 501–659 nm but inclusion of 532 nm. The centrifuge tube filled with the PEA‐CuS NPs was placed in a water tank. Water acted as a coupling medium, and its temperature remained constant at 23 °C during the studies. A right angle prism guided the beam to fall on a 1.5 mL centrifuge tube loaded with the PEA‐CuS NPs. An immersion‐type unfocused ultrasound transducer (Panametrics, V320‐SU), having a center frequency of 7.5 MHz, 70% fractional bandwidth, and 10 mm diameter, was used to detect radio frequency (RF) signals. The obtained PA signals were then amplified through a pulser/receiver module (JSR, DPR300). Finally, a data acquisition (DAQ) card (Adlink PCIe‐9852) was used to digitize the PA signals at a sampling frequency of 50 MHz and save the RF lines, each having 8000 data points. The trigger signal from the laser system was employed to synchronize the DAQ system. For an individual PEA‐CuS NPs system, 300 RF lines were recorded at each optical exposure for further analysis. Optical wavelengths from 350 nm to 1100 nm were utilized for the experiments.

To perform the PA studies of PEA‐CuS NPs, six different concentrations of 350, 175, 87.5, 43.75, 21.875, and 10.93 μg mL^−1^ were prepared. Initially, the PA spectra were measured in the optical wavelength from 350 to 1100 nm, with a spacing of 10 nm. Subsequently, the concentration‐dependent amplitude of the PA signals was monitored for all samples at 532 and 690 nm optical wavelengths. The laser pulse energy was fixed to 42.35 and 30.41 mJ at 532 and 690 nm, respectively. Furthermore, we investigated the PA responses of these samples when the energy of the incident laser beam varied from 13 to 34 mJ at 690 nm wavelength.

### 2.8 Statistical Analysis

All results and measurements were shown as mean ± SD. Comparisons between the mean values of the different groups were analyzed by the unpaired two‐tailed Student's *t*‐test (using the *t*‐test calculator, GraphPad), where (*) p < 0.05 was considered statistically significant and (**) p < 0.01 was considered highly statistically significant.

## 3 Results and Discussion

The highly water‐dispersible PEA‐CuS NPs were synthesized through a single step soft chemical approach using PEA as a surface passivating agent. PEA is an ethanolamine derivative usually used to construct phospholipids. It has two different functional groups, such as amine and phosphate. The phosphate group of the PEA molecule has a high coordinative affinity toward sulfide NPs, leaving the amine groups freely exposed to the aqueous medium for providing water dispersibility to them. The aqueous colloidal stable green covellite CuS phase was synthesized by reacting an aqueous solution of Cu^2+^ ions with Na_2_S in the presence of the PEA at 80 °C. The stable green CuS NPs were successfully prepared with a molar ratio Cu^2+^: S^2−^: PEA of 1:3:3. The use of high concentrations of S^2−^ ions (with respect to Cu^2+^ ions) in the preparation of the stable PEA‐CuS NPs was decided based on our previous study.^[^ ^24^ ^]^ It has been found that the green CuS phase formed at low concentration of S^2−^ ions converted into Cu^2+^ ions (forming a bluish color solution) within 3–4 days in the solution phase. Based on the thermodynamic aspect, this reverse reaction was favored due to the dissociation of the ultrasmall particles (size less than critical radius of stable CuS NPs) formed into their ionic form. However, the high concentration of S^2−^ ions favors the growth of nanoparticles beyond the critical radius, like the seed‐mediated growth process.

**Figure 1:**
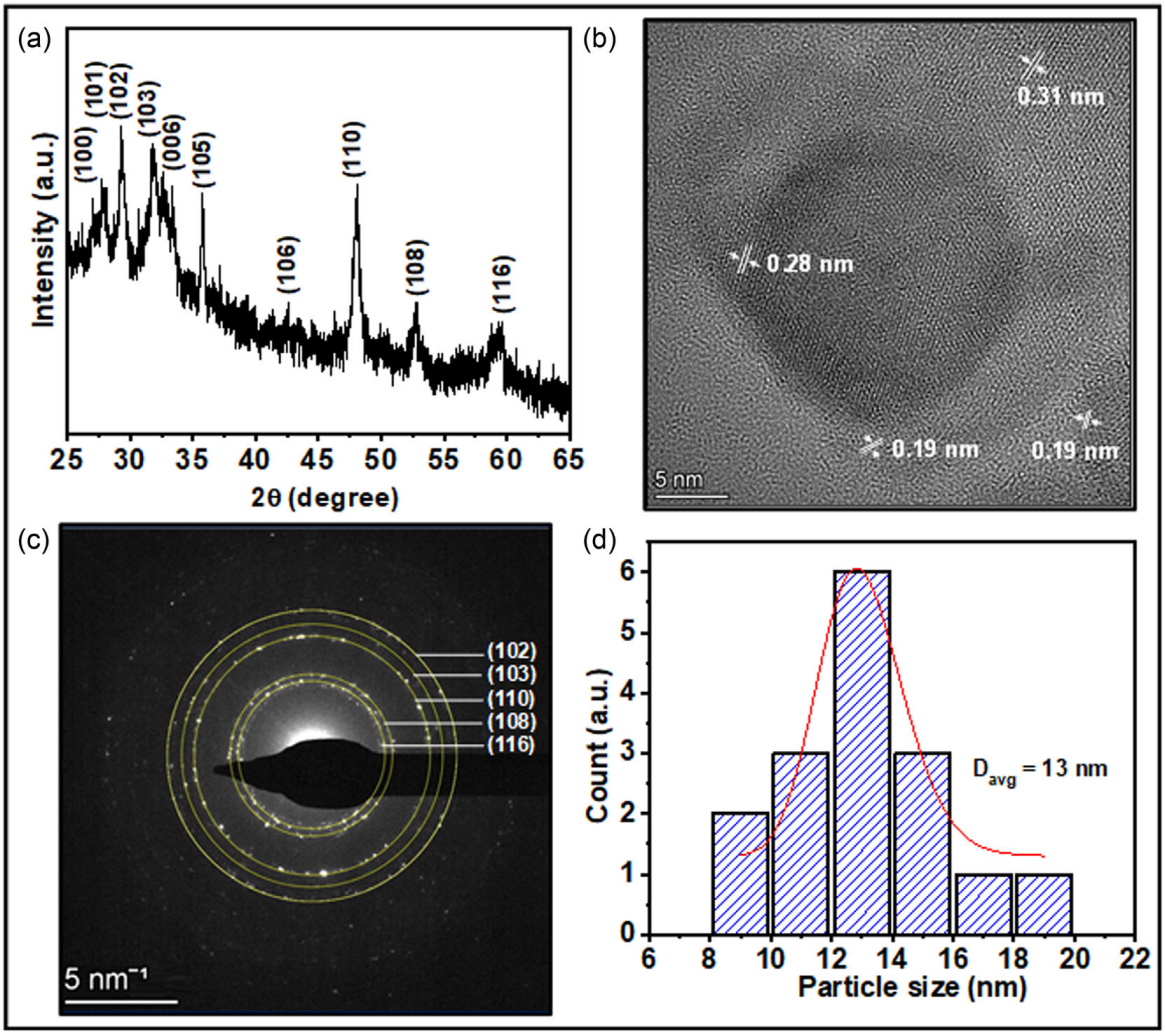
a) XRD pattern, b) HRTEM image, c) SAED pattern, and d) particle size distribution plot of the PEA‐CuS NPs.

**Figure** **1** shows the (a) XRD pattern, (b) HRTEM image, (c) SAED pattern, and (d) the particle size distribution plot of the PEA‐CuS NPs. The appearance of the well‐defined characteristic diffraction peaks in the XRD pattern of the PEA‐CuS NPs (Figure 1a) corresponding to the (100), (101), (102), (103), (006), (105), (106), (110), (108), and (116) lattice planes suggested the formation of hexagonal covellite CuS phase (matches well with the JCPDS Card No. 06‐0464 of CuS).^[^ ^42^ ^]^ Further, the presence of a broad peak with background noise indicated the nanocrystalline nature of the PEA‐CuS NPs. High resolution TEM (HRTEM) micrograph (Figure 1b) showed the presence of various lattice fringes. Specifically, the interplanar spacing of 0.31 nm, 0.28 nm, and 0.19 nm were indexed to the (102), (103), and (110) planes of covellite CuS, respectively (fast Fourier transform [FFT], inverse FFT [IFFT], and the plot profile of the PEA‐CuS NPs used for determining d spacing are provided in Figure S1, Supporting Information). Furthermore, the diffraction ring from the selective area electron diffraction (SAED) pattern of the PEA‐CuS NPs (Figure 1c) confirmed the formation of a highly‐crystalline covellite phase of the CuS NPs. The particle size distribution (Figure 1d) obtained from the TEM micrograph (TEM image of PEA‐CuS NPs used for obtaining size distribution plot is shown in Figure S2, Supporting Information) showed the formation of nanosized CuS NPs ranging from 6–20 nm, with an average size of 13 nm.

**Figure 2:**
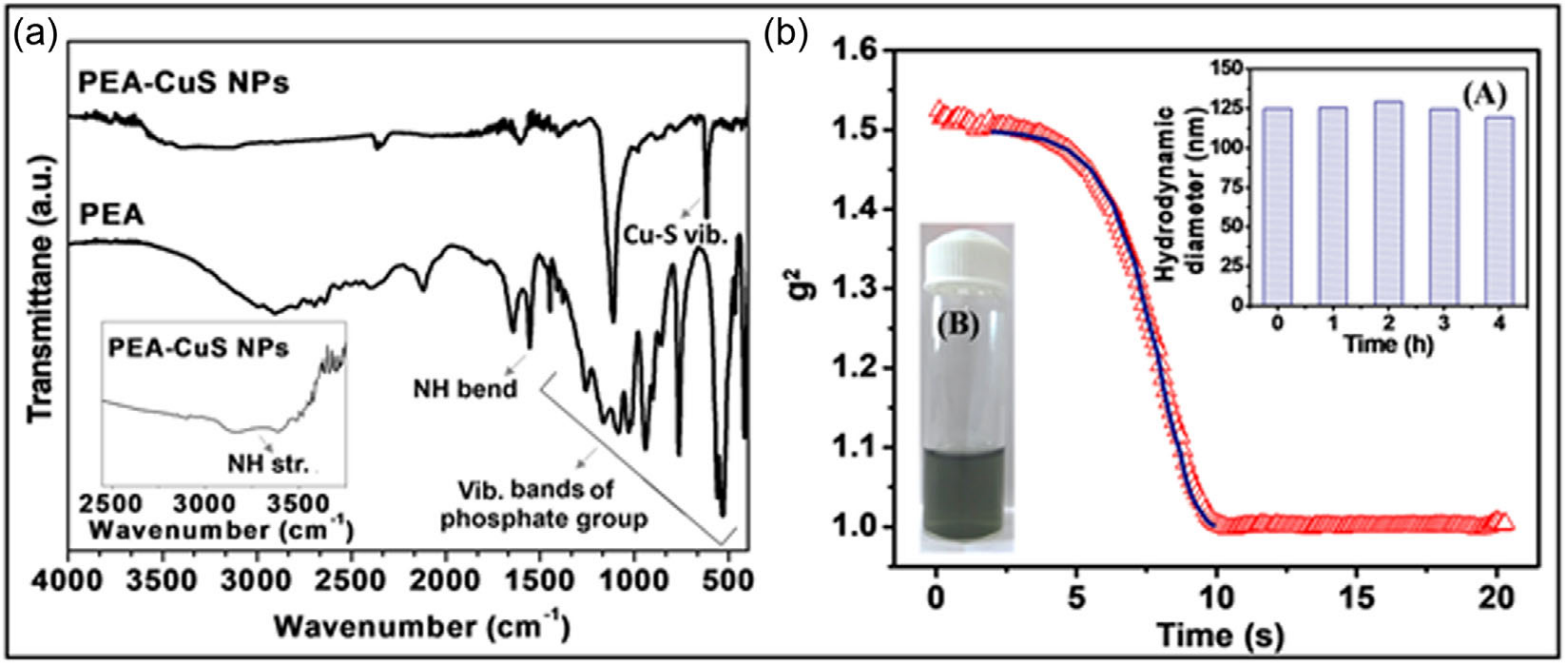
a) FTIR spectra of the PEA and PEA‐CuS NPs (inset: magnified spectrum of the PEA‐CuS NPs within the region 2450–3750 cm^−1^) and b) autocorrelation function of aqueous suspension of PEA‐CuS NPs (inset A: variation of H_D_ as a function of time; inset B: photograph of aqueous colloidal dispersion of the PEA‐CuS NPs).

**Figure** **2** shows the (a) FTIR spectra of the PEA and the PEA‐CuS NPs (Inset: magnified spectrum of the PEA‐CuS NPs in the region 2450–3750 cm^−1^) and (b) autocorrelation function of the aqueous suspension of the PEA‐CuS NPs (Inset A: variation of H_D_ as a function of time; Inset B: photograph of aqueous colloidal dispersion of high‐resolution TEM he PEA‐CuS NPs). The FTIR bands for the PEA are well‐resolved, whereas those for the PEA‐CuS NPs are rather broad and few (Figure 2a). In the FTIR spectrum of the PEA, characteristic bands corresponding to the phosphate stretching vibrations of P=O, PO_2_^−^, and PO_3_^−^ groups are observed in the region 800–1250 cm^−^^1^.^[^ ^43^, ^44^ ^]^ Additionally, the bands at 765 cm^−^^1^ and 560 cm^−^^1^ are attributed to the O‐P‐O bending mode. The FTIR spectra of the PEA and the PEA‐CuS NPs in the region 1500–400 cm^−1^ with corresponding band assignments is shown in Figure S3, Supporting Information. However, most of these vibrational modes appeared in the FTIR spectrum of the PEA‐CuS NPs with slight shifting in their band position. In the present study, a very sharp and intense band was observed at ≈1118 cm^−1^. The FTIR spectrum of the PEA‐CuS NPs can be assigned to the P–O^−^ groups. Most probably, the coordinative coupling of PEA with the CuS NPs through the oxygen atoms of the phosphate group render predominate P–O^−^ moieties to the surface of the PEA‐CuS NPs. Moreover, the vibrational band associated with P–O–M (M = metal) is also reported to appear at around 1100 cm^−1^. For instance, Lindner et al. reported P–O–Si band at ≈1100 cm^−1^ in P/Al‐doped silica glass.^[^ ^45^ ^]^ Thus, it is difficult to distinguish P—O—Cu bond vibrations in the present study as it may lie in the same region. Further, the broad band in the region 3050–3650 cm^−^^1^ and vibrational mode at ≈1550 cm^−1^ in the FTIR spectrum of the PEA‐CuS NPs (Figure 2a and its inset) can be attributed to the stretching and bending vibrations of the amine (NH_2_) groups of the PEA. In addition, the appearance of a new vibrational mode at ≈619 cm^−1^ in the FTIR spectrum of the PEA‐CuS NPs can be ascribed to the stretching vibration of the Cu—S bond.^[^ ^24^ ^]^ The FTIR results confirmed the formation of the CuS phase and the conjugation of the PEA to the CuS NPs via the phosphate moiety, leaving the amine groups free.

**Figure 3:**
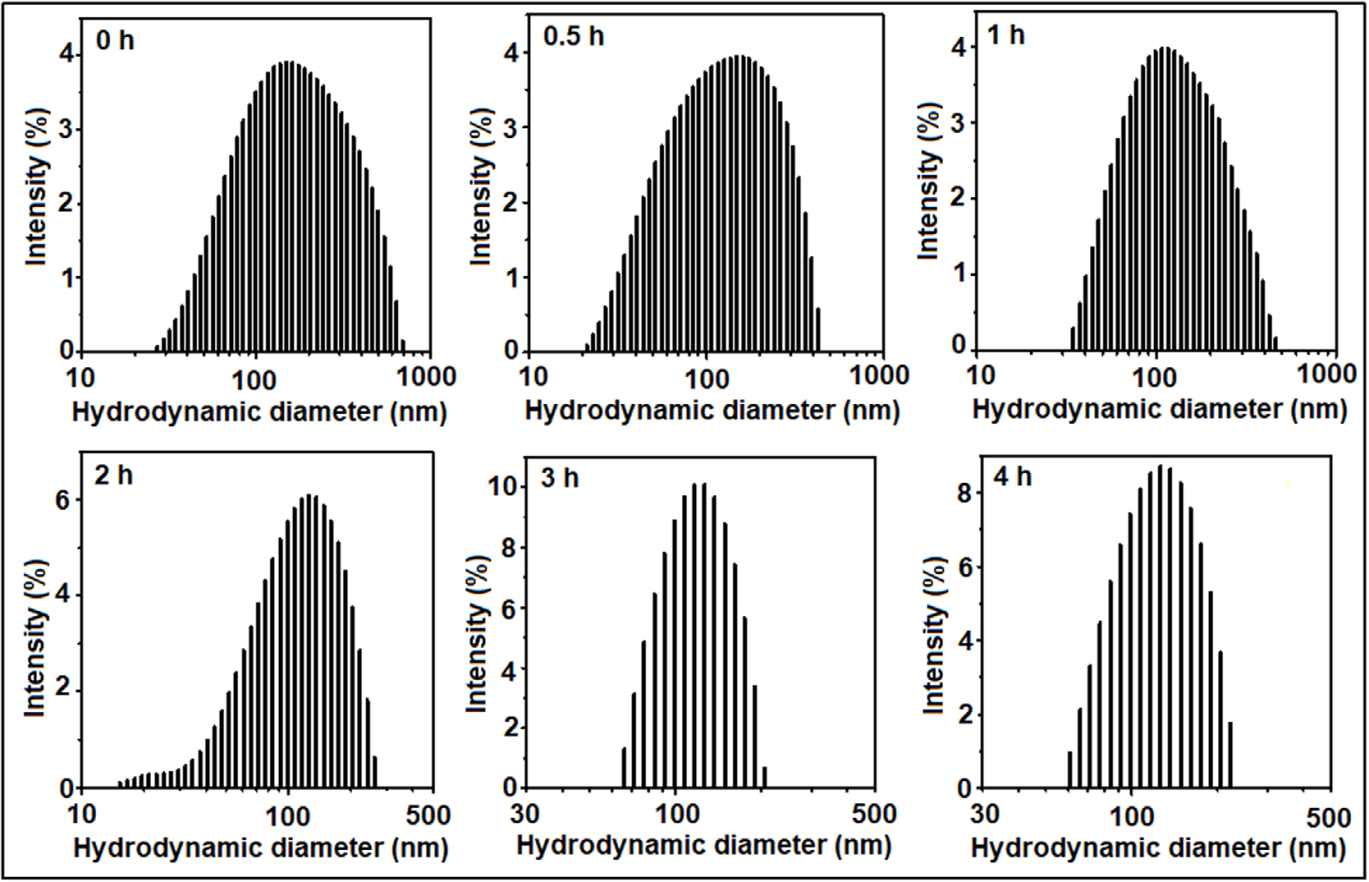
DLS plots showing intensity weighted H_D_ distribution of the aqueous suspension of the PEA‐CuS NPs at different times.

For using the NPs in biomedical application, their aqueous colloidal stability is of utmost important. Therefore, colloidal stability studies of the PEA‐CuS NPs were performed using dynamic light scattering (DLS). The exponential decay of the intensity‐weighted autocorrelation function indicates a mean H_D_ of 120 nm, with a polydispersity index (PDI) of 0.25, signifying good colloidal stability (Figure 2b). These nanoparticles exhibited hydrophilic behavior due to hydrogen bonding between their surface functional groups (freely exposed NH_2_) and water molecules. This hydrophilicity likely contributes to the larger particle size observed compared to the TEM analysis. Moreover, the inherent polydispersity and propensity for self‐aggregation in the nanoparticles likely contribute to the observed larger average size, as DLS inherently favors measurements weighted toward larger particle populations. Despite this, the intensity‐weighted H_D_ distribution exhibits negligible variation over time (Inset of Figure 2b and **Figure** **3**), underscoring the exceptional colloidal stability of the nanoparticles in aqueous media.^[^ ^24^ ^]^ Furthermore, the pH‐dependent ZP measurements of the aqueous suspension containing the PEA‐CuS NPs showed charge reversal behavior with a zero point charge (pH_zpc_) around 4 (Figure S4, Supporting Information). The ZP varied from +6 to −38 mV at a pH ranging from 2 to 12. This charge reversal is mainly due to the formation of protonated NH_3_^+^ at pH < pH_zpc_, whereas at pH > pH_zpc_, an excess of OH^−^ ions result in the formation of NH_2_OH^−^ moiety, which gives a negative ZP.^[^ ^46^ ^]^ The ZP at pH 7 is found to be around ‐17.5 mV, which provides additional aqueous colloidal stability to the PEA‐CuS NPs through electrostatic repulsion in supplement to hydrogen bonding. Based on the FTIR and light scattering studies, it has been presumed that the introduction of PEA molecules onto the surface of the CuS NPs leads to the formation of PEA‐CuS NPs by the conjugation of phosphate groups with the CuS NPs, either using two or three of its oxygen molecules (schematic representation revealing probable Cu–phosphate coordinative coupling shown in Figure S5, Supporting Information). The present study strongly supports our earlier work on the development of crystallographic (covellite CuS phase) and aqueous colloidally stabilized LP‐CuS NPs using a phosphate ligand (a linear polyphosphate molecule, sodium tripolyphosphate) as a surface passivating agent.^[^ ^24^ ^]^ This opens up a new avenue of utilizing phosphate‐based ligands for phase stabilization of sulfide nanostructures other than the commonly used thiol ligands.

**Figure 4:**
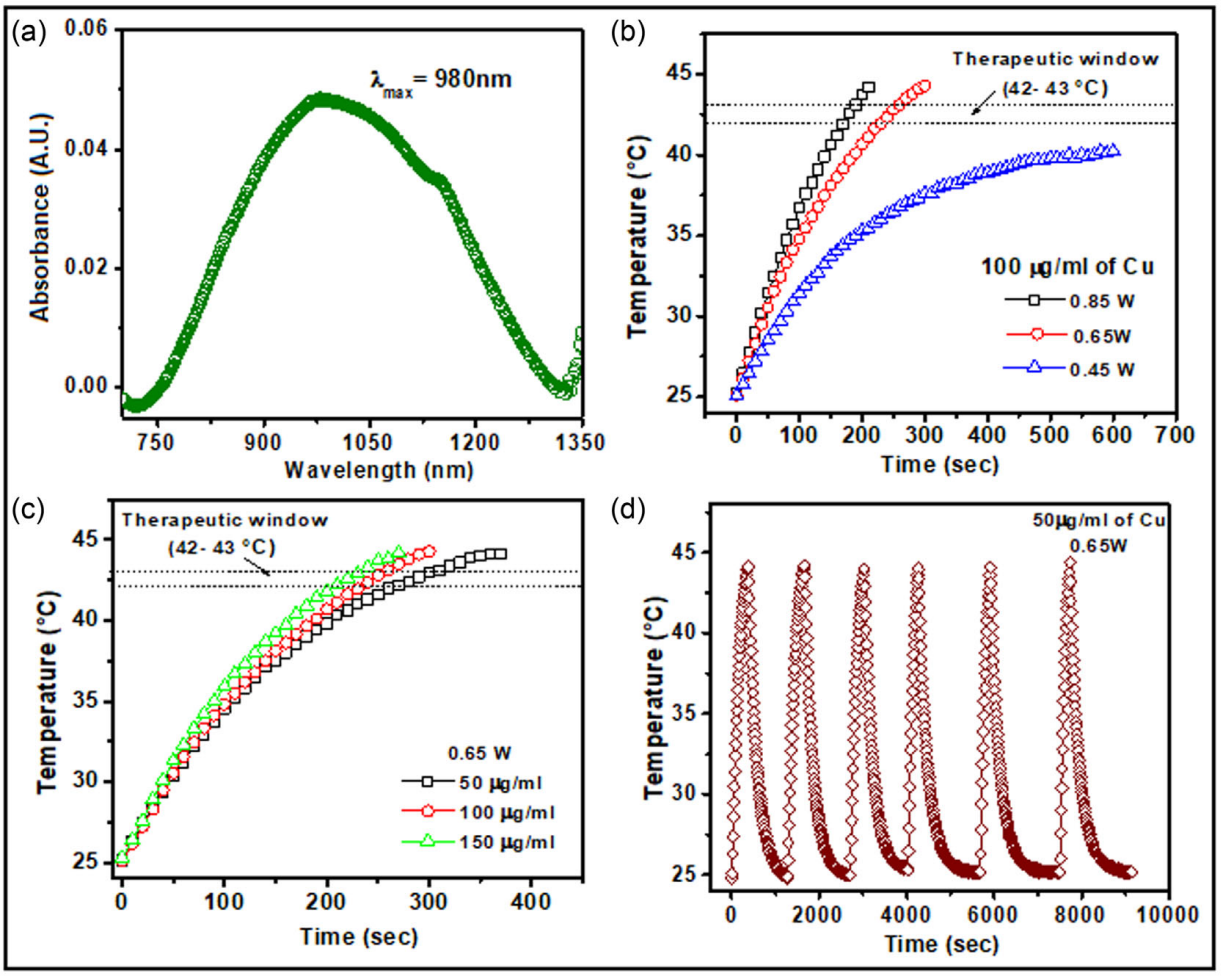
a) Visible–NIR absorption spectrum of the aqueous dispersion of the PEA‐CuS NPs, and photothermal heating efficacy of the aqueous suspension of the PEA‐CuS NPs under 980 nm laser light irradiation at b) different laser power containing 100 μg mL^−1^ of Cu, c) different concentrations at a fixed power of 0.65 W, and d) recycling capability of 50 μg mL^−1^ Cu at a laser power of 0.65 W up to six cycles.

**Figure** **4** shows (a) visible–NIR absorption spectrum of aqueous dispersion of the PEA‐CuS NPs, and the photothermal heating efficacy of the aqueous suspension of the PEA‐CuS NPs under 980 nm laser light irradiation at (b) different laser powers containing 100 μg/mL of Cu, (c) different concentration at a fixed power of 0.65 W, and (d) recycling capability of 50 μg/mL Cu at a laser power of 0.65 W up to six cycles. The absorption spectrum (Figure 4a) demonstrates a pronounced peak in the NIR region (750–1350 nm), attributable to the localized surface plasmon resonance (SPR) of valence‐band free carriers (positive holes) in covellite CuS.^[^ ^47^ ^]^ The maximum absorbance of the PEA‐CuS NPs is centered at ≈980 nm, making 980 nm laser irradiation optimal for evaluating the photothermal heating efficacy of the PEA‐CuS NPs over our earlier reported LP‐CuS NPs (whose maxima lies at ≈900 nm) by fully exploiting their SPR properties. The pronounced SPR absorption in the NIR region motivated the investigation of the photothermal performance of the PEA‐CuS NPs. As shown in Figure 4b, the time required to achieve the therapeutic temperature range (42–43 °C) for a suspension containing 100 μg mL^−1^ of Cu is 240 sec (4 min) at a laser power of 0.65 W and 180 sec (3 min) at 0.85 W. In contrast, the therapeutic temperature was not reached at a laser power of 0.45 W even after 600 sec (10 min). However, it is noteworthy to mention that the time required to reach the therapeutic range for PEA‐CuS NPs is lower than that reported for LP‐CuS NPs.^[^ ^24^ ^]^ For instance, PEA‐CuS NPs (100 μg mL^−1^ of Cu) took only 4 min to achieve a therapeutic temperature of 42–43 °C with laser power of 0.65 W, whereas that of LP‐CuS NPs required 6.5 min. The observed higher heating rate of PEA‐CuS NPs may be attributed to its strong NIR absorption capability with maxima very closed to 980 nm and the use of small PEA molecules as surface passivating agents over linear polyphosphate. Furthermore, as illustrated in Figure 4c, the time required to reach the therapeutic temperature window decreases with increasing Cu concentration in the aqueous suspension. These results indicate that the time to achieve the therapeutic window is strongly dependent on both the laser power and the concentration of nanoparticles in the suspension. To confirm that the observed heating effect originates primarily from the presence of the PEA‐CuS NPs, a control experiment was conducted using 1 mL of DI water irradiated with a 980 nm laser at a power of 0.65 W. The temperature of the water increased only to ≈37 °C after 20 min, highlighting that the temperature rise observed in the aqueous suspension of the PEA‐CuS NPs is predominantly due to CuS (Figure S6, Supporting Information). An ideal photothermal agent should exhibit excellent NIR photostability. To evaluate the NIR photostability of the PEA‐CuS NPs, an aqueous suspension was subjected to cyclic irradiation with a 980 nm laser at a power of 0.65 W, followed by natural cooling to room temperature in the absence of laser irradiation. This heating–cooling cycle was repeated six times, and the photothermal stability results are presented in Figure 4d. The reusability of the PEA‐CuS NPs was demonstrated over six consecutive cycles, with negligible loss in heating efficiency, indicating exceptional photothermal stability. This robust stability, combined with a consistent photothermal performance, highlights the potential of the PEA‐CuS NPs for repeated applications in PTT.

**Figure 5:**
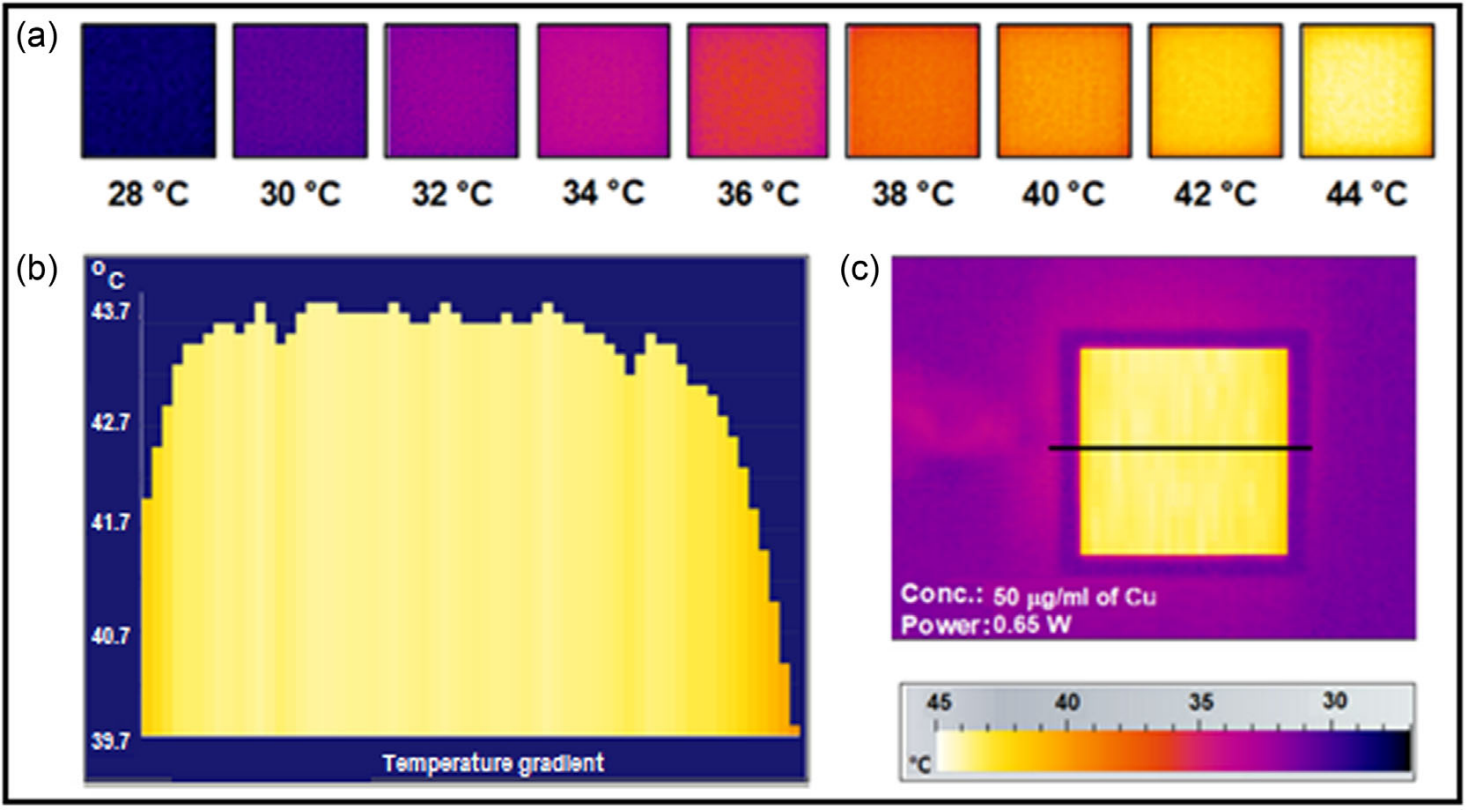
a) IR thermograms showing an increase in the temperature of the aqueous dispersion of the PEA‐CuS NPs under 980 nm laser light irradiation and b) temperature‐gradient plot obtained across the drawn line of the IR thermogram shown in c).

IR thermogram provides compelling evidence of the photothermal heating activity of the PEA‐CuS NPs (**Figure** **5**). The IR thermogram of a 50 μg mL^−1^ Cu dispersion of PEA‐CuS NPs in DI demonstrates a distinct localized heating effect. Regions depicted in blue represent temperatures near 27 °C, while areas in bright yellow correspond to a significant temperature increase, reaching ≈44 °C. Moreover, the temperature gradient observed along a defined line in the thermogram emphasizes the spatially localized nature of the heating effect induced by the nanoparticles. This precise localized heating is of paramount importance, as it minimizes collateral thermal damage to the surrounding healthy cells, thereby enhancing the therapeutic potential of the PEA‐CuS NPs in PTT.

**Figure 6:**
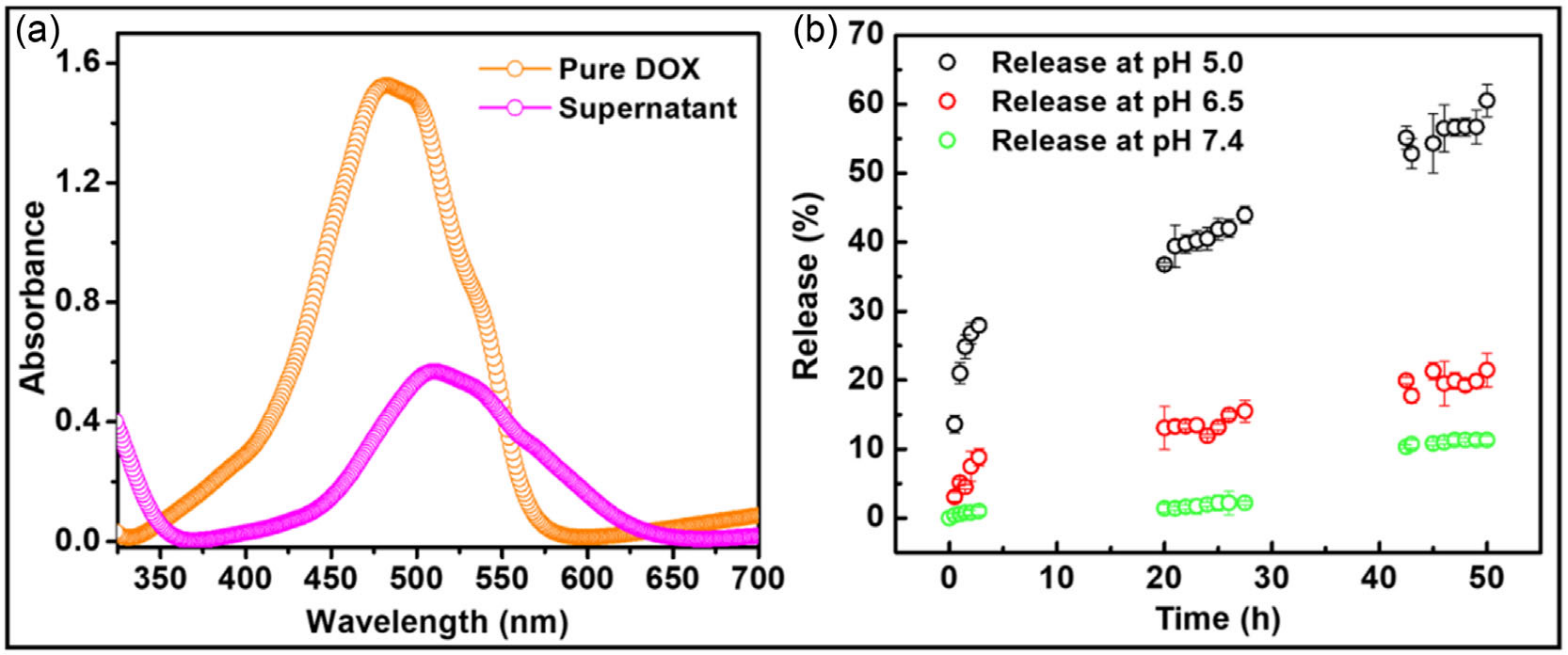
a) UV–vis absorption plots of the pure DOX and supernatant solution obtained after separation of DOX‐loaded PEA‐CuS NPs and b) pH‐dependent release profile of the drug from DOX@PEA‐CuS NPs under reservoir‐sink condition at 37 °C.

The broad‐spectrum anticancer drug, DOX was utilized to assess the potential of the PEA‐CuS NPs as a drug delivery vehicle. DOX is a positively charged drug. UV–vis absorbance studies (**Figure** **6**a) revealed a maximum loading efficiency of 55% at a DOX‐to‐particle ratio of 1:10 (5 mg of particles and 0.5 mg of DOX). The positively charged drug DOX (ZP = +9 mV, Figure S7, Supporting Information) interact electrostatically with the negatively charged PEA‐CuS NPs having ZP of −17 eV at neutral pH. Moreover, there have been various reports showing the electrostatic interaction between positively charged DOX and negatively charge nanocarriers.^[^ ^24^, ^48^, ^49^ ^]^ The pH‐dependent drug release from DOX@PEA‐CuS NPs was evaluated under reservoir–sink conditions at 37 °C (Figure 6b). It was observed that ≈60% of the loaded drug was released slowly over a period of 50 h at pH 5.0. In acidic medium, the negatively charged NH_2_OH^−^ moieties of the PEA‐CuS NPs get partially neutralize by H^+^ ions, resulting in the weakening of the electrostatic interaction between the drug and the PEA‐CuS NPs. Thus, the observed higher release of DOX from the DOX@PEA‐CuS NPs can be attributed to the gradual weakening or breaking of the electrostatic interactions between the particles and DOX at a lower pH.^[^ ^24^, ^48^ ^]^ Additionally, at pH 6.5 and 7.4, the percentage of drug released decreased to 20% and 10%, respectively. The significantly low release of drug at pH 7.4 indicates the good stability of the drug formulation under physiological medium. This controlled and sustained release profile suggests that the developed drug‐loaded system holds significant potential as a long‐term therapeutic platform.

The synthesized PEA‐CuS NPs demonstrates significant potential for PTT due to their efficient conversion of optical energy into thermal energy. Additionally, the controlled drug release at acidic pH from drug‐loaded NPs makes them suitable for chemo–photothermal therapy applications. In vitro biocompatibility of the PEA‐CuS NPs was evaluated using the WI26VA4 normal lung cell line. Cells were treated with varying concentrations of the PEA‐CuS NPs for 24 h at 37 °C in complete media. The results indicated that ≈80% of the cells remained viable across all tested concentrations, highlighting the excellent biocompatibility of the PEA‐CuS NPs (Figure S8, Supporting Information).

**Figure 7:**
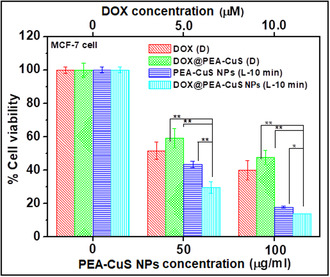
Viability of MCF‐7 cells upon incubation with the PEA‐CuS NPs and the DOX@PEA‐CuS NPs under irradiation of NIR light (L‐10 min) and dark (D) conditions. Data represent the mean ±SD (*n* = 3); the statistically significant values were obtained using a student *t*‐test, **p < 0.01.

**Figure 8:**
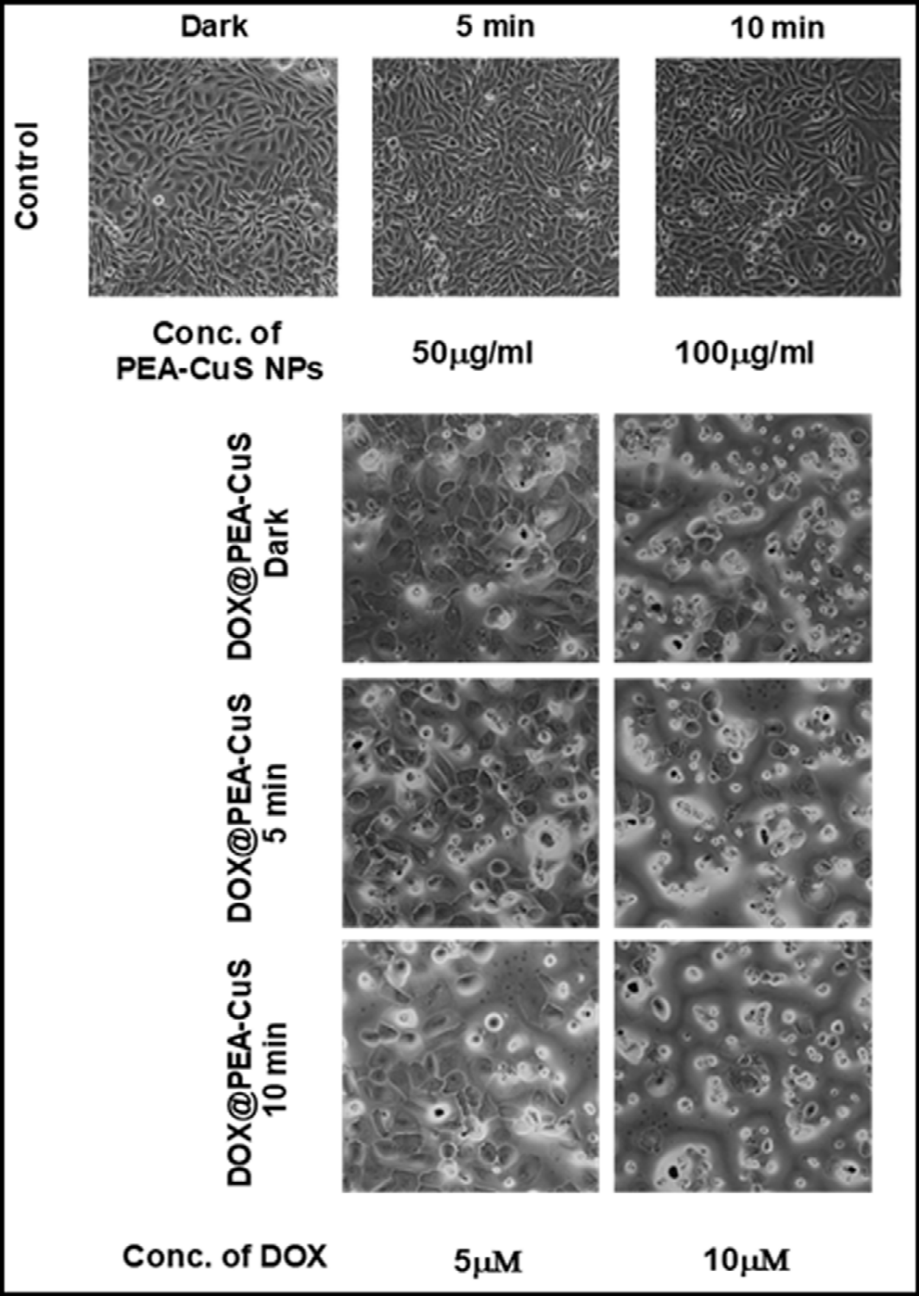
Bright‐field imaging of the MCF‐7 cells with treatment of PEA‐CuS NPs and DOX@PEA‐CuS NPs under dark and NIR laser (980 nm, 0.65 W) exposer for 5 and 10 min.

Cytotoxicity studies were conducted on the MCF‐7 cancer cells to assess the photothermal and chemo‐photothermal efficacy of the developed system. Treatments included DOX, DOX@PEA‐CuS NPs under dark conditions, and PEA‐CuS NPs and DOX@PEA‐CuS NPs under laser irradiation (**Figure** **7** and Figure S9, Supporting Information). Under dark conditions, DOX@PEA‐CuS NPs exhibited lower cytotoxicity compared to free DOX, and both systems demonstrated dose‐dependent toxicity. Notably, treatment with PEA‐CuS NPs under laser irradiation (5 and 10 min) resulted in a significant reduction in cell viability, demonstrating the photothermal effect. A combination therapy with the DOX@PEA‐CuS NPs under 10 min NIR irradiation further enhanced cytotoxicity, achieving over 80% cell death at a concentration of 100 μg mL^−1^ of the DOX@PEA‐CuS NPs containing 10 μM of DOX. This enhanced cytotoxicity was corroborated by bright field imaging, which revealed a marked reduction in cell population with increasing concentrations of the PEA‐CuS NPs and DOX@PEA‐CuS NPs under NIR exposure (**Figure** **8**). The observed toxicity is attributed to the heightened sensitivity of the cancer cells to DOX in the presence of NIR irradiation. These findings prompt the potential of the PEA‐CuS NPs as a robust platform for combined photothermal and chemo‐photothermal cancer therapy.

**Figure 9:**
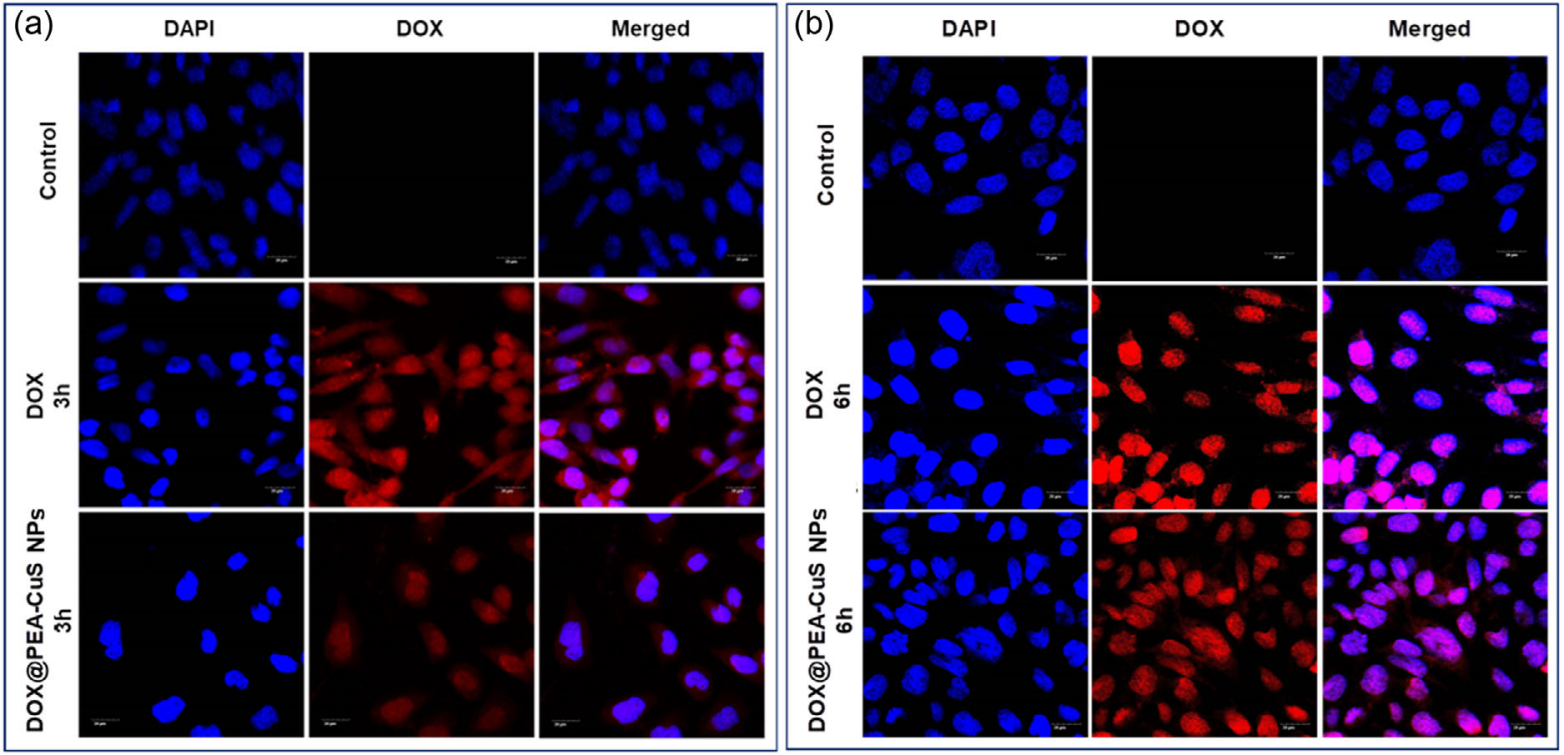
CLSM images of the MCF‐7 cells after incubation with DOX and DOX@PEA‐CuS NPs (at a DOX concentration of 5 μM) under culture conditions for a) 3 h and b) 6 h (DAPI was used for nuclear staining, red laser for DOX, blue laser for DAPI, scale bar: 15 μm).

For a drug‐loaded system, achieving high therapeutic efficacy is essential. To evaluate intracellular drug delivery, the cellular uptake of the DOX@PEA‐CuS NPs and pure DOX was examined in the MCF‐7 cancer cells using CLSM. **Figure** **9** shows the CLSM images of the MCF‐7 cells after incubation with DOX and DOX@PEA‐CuS NPs (at a DOX concentration of 5 μM) under culture conditions for (a) 3 h and (b) 6 h (DAPI was used for nuclear staining, red laser for DOX, blue laser for DAPI, scale bar: 15 μm). Untreated cells were used as a negative control, with only autofluorescence observed. Nuclei were stained with DAPI, a nuclear staining agent, emitting blue fluorescence. Red fluorescence from the DOX was clearly detected in both pure DOX and the DOX@PEA‐CuS NPs, indicating time‐dependent cellular internalization. The merged images of DAPI and DOX fluorescence yielded magenta fluorescence, confirming nuclear localization of DOX. For pure DOX, the fluorescence overlap with DAPI signals demonstrated predominant nuclear internalization. In contrast, the DOX@PEA‐CuS NPs exhibited fluorescence in both the cytoplasm and nucleus, signifying dual localization of DOX. Time‐dependent imaging showed increased nuclear accumulation of DOX over time. Previous studies suggest that pure DOX rapidly internalizes into the cell membrane and nucleus via passive diffusion, whereas nanoparticles loaded with drugs are internalized through endocytosis.^[^ ^50^ ^]^ The observed increase in nuclear localization of DOX over time suggests that the DOX‐loaded nanoparticles are passively targeted to tumors through the enhanced permeability and retention (EPR) effect. The nanoparticles are initially internalized into the cytoplasm via endocytosis, followed by a gradual release of DOX into the nucleus. This mechanism underscores the potential of the DOX@PEA‐CuS NPs for efficient intracellular drug delivery and sustained therapeutic action.

**Figure 10:**
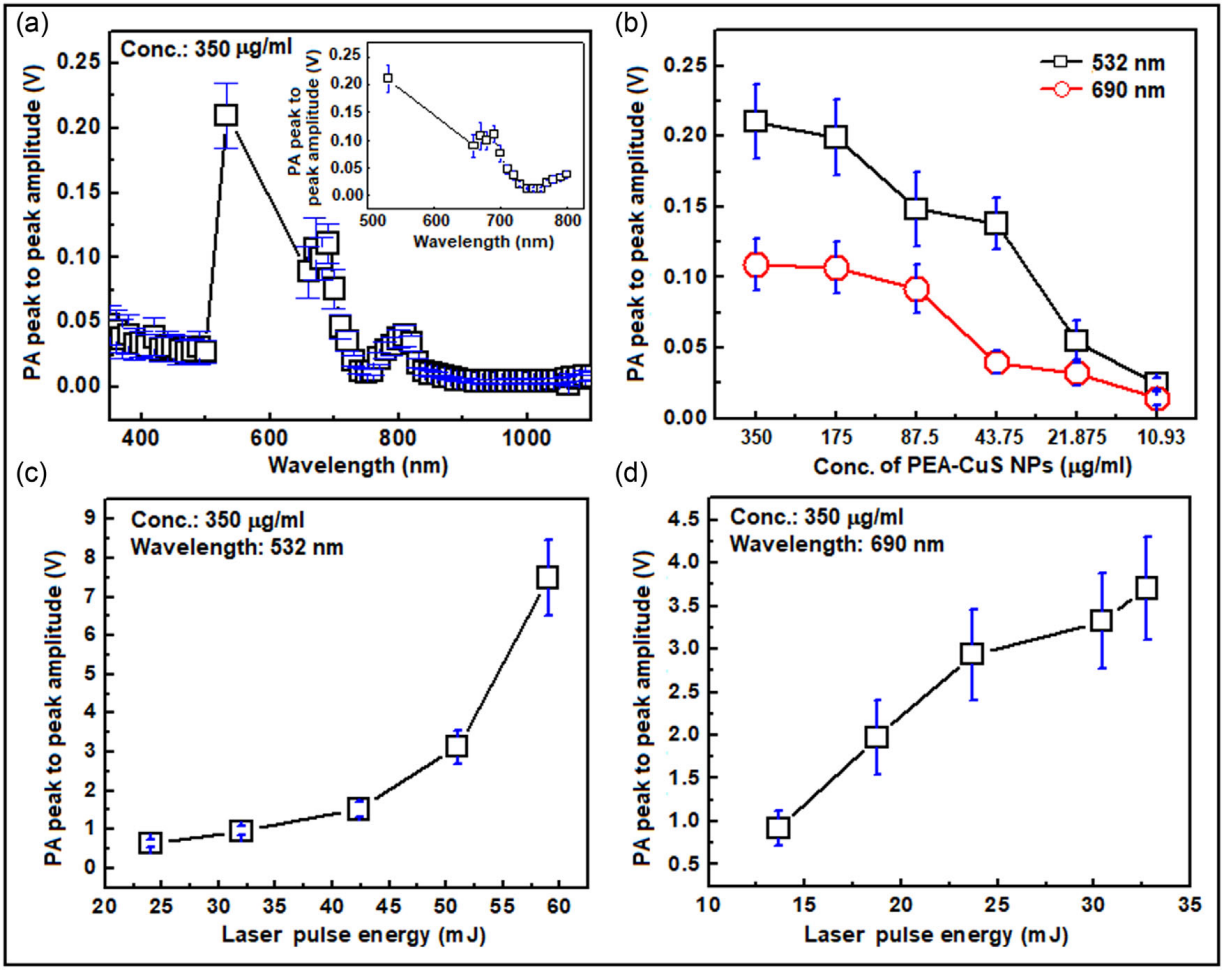
Plots of PA peak‐to‐peak amplitude for a) 350 μg mL^−1^ of PEA‐CuS NPs with incident optical wavelength in the range of 350–1100 nm (inset: its PA peak‐to‐peak amplitude in the range of 500–800 nm), b) different concentrations of PEA‐CuS NPs at wavelengths 532 and 690 nm, and variation of PA peak‐to‐peak amplitude at 350 μg mL^−1^ of PEA‐CuS NPs with different laser pulse energy at c) 532 nm and d) 690 nm.

The PA wave generation ability of the PEA‐CuS NPs was also investigated for image‐guided chemo–photothermal therapy. The PA spectra (in the range of 350 to 1100 nm) has shown significantly low and fluctuating signals in the range of 350 to 490 nm, whereas almost no signal in the range of 910 to 1100 nm. The spectral amplitudes were found to be maximum at 532 nm at the concentration 350 μg mL^−1^. The secondary peaks are observed at 690 and 800 nm respectively, where the height of each peak shows ≈50% decrease compared to that of the previous peak (**Figure** **10**a). The concentration‐dependent PA peak‐to‐peak amplitude showed an almost linear variation at 532 nm as well as at 690 nm, respectively (Figure 10b). The PA signal strength monotonically decreased with decreasing sample concentration from 350 to 10.93 μg mL^−1^. We have also carried out the laser pulse energy dependence at two different laser wavelengths (532 and 690 nm) for the PA peak‐to‐peak amplitude (Figure 10c,d). It was observed that the PA peak‐to‐peak amplitude increases linearly at both 532 and 690 nm. These findings demonstrated that the PEA‐CuS NPs possess good PA signal. Specifically, the present study demonstrated the development of water‐dispersible and biocompatible PEA‐CuS NPs for combinatorial chemo–photothermal therapy along with PA ability.

## 4 Conclusion

In summary, we have successfully constructed a chemo–photothermal therapeutic system based on the DOX‐conjugated CuS NPs, which is surface functionalized using a phosphorylethanolamine molecule. The pH‐dependent zeta potential has provided the base for DOX loading and their subsequent release in aid of the photothermal effect produced by the CuS under NIR irradiation in mildly acidic environments, which is beneficial for improving the effect of the chemo–photothermal therapy. In addition, the as‐prepared CuS NPs show good water dispersibility and favorable biocompatibility required for efficient use in biomedical application. In comparison to individual counterparts, the drug‐loaded system demonstrated significant cellular internalization into the MCF‐7 cells and significantly greater toxicity towards them upon NIR irradiation (980 nm). Additionally, with pulse laser stimulation (532 and 690 nm), these PEA‐CuS NPs have demonstrated outstanding PA characteristics. Thus, the developed nanoformulation may be used as a viable nanosystem for image‐guided chemo–photothermal therapy.

## Conflict of Interest

The authors declare no conflict of interest.

## Supporting information

Supplementary Material

## Data Availability

The data that support the findings of this study are available from the corresponding author upon reasonable request.
