# Supplementary material for: Development of Water‐Dispersible CuS Nanoparticles for Chemo–Photothermal Therapy and Photoacoustic Application
Source: Chemphyschem. 2025 Jul 3;26(16):e202500016. doi: 10.1002/cphc.202500016 (PMC12388176; doi:10.1002/cphc.202500016)
Supplement: Supplementary file 1 — Supplementary Material [file CPHC-26-e202500016-s001.pdf]

Supporting information

Development of Water-dispersible CuS Nanoparticles for Chemo-photothermal Therapy and Photoacoustic Application

Sonali Gupta[a,b], Bijaideep Dutta[a,b], Subhadip Paul[c], Ratan K. Saha[c], Kanhu C. Barick[a,b]* and Puthusserickal A. Hassan[a,b]*

[a]*Chemistry Division, Bhabha Atomic Research Centre, Trombay, Mumbai-400085, India*
[b]*Homi Bhabha National Institute, Anushaktinagar, Mumbai - 400094, India*
[c]*Department of Applied Sciences, Indian Institute of Information Technology Allahabad, Allahabad-211012, India*

**E-mail: hassan@barc.gov.in, kcbarick@barc.gov.in, Tel.: + 91 22 2559 0284*

Figures detail:

Figure S1. (A) HRTEM image of PEA-CuS NPs showing interplanar spacing (marked at different region) and (B) their respective FFT (first column), IFFT (second column) and profile plot (third column) along marked line in IFFT image.

Figure S2. TEM image of PEA-CuS NPs used for obtaining size distribution plot.

Figure S3. FTIR spectra of PEA and PEA-CuS NPs in the region of 1500-400 cm^{-1} with corresponding band assignments.

Figure S4. pH dependent zeta-potential of aqueous suspension of PEA-CuS NPs.

Figure S5. Schematic representation revealing probable Cu-phosphate coordinative coupling of PEA-CuS NPs.

Figure S6. Heating profile of 1ml of DI water under 980 nm (NIR irradiation) laser of power of 0.65 W.

Figure S7. Zeta potential plot of aqueous solution of pure DOX

Figure S8. Cell viability of WI26VA4 with treatment of PEA-CuS NPs for 24 h at 37 ˚C and 5% CO_2 under cell culture conditions.

Figure S9. Viability of MCF-7 cells upon incubated with PEA-CuS NPs and DOX@PEA-CuS NPs under irradiation of NIR light (L-5 min).

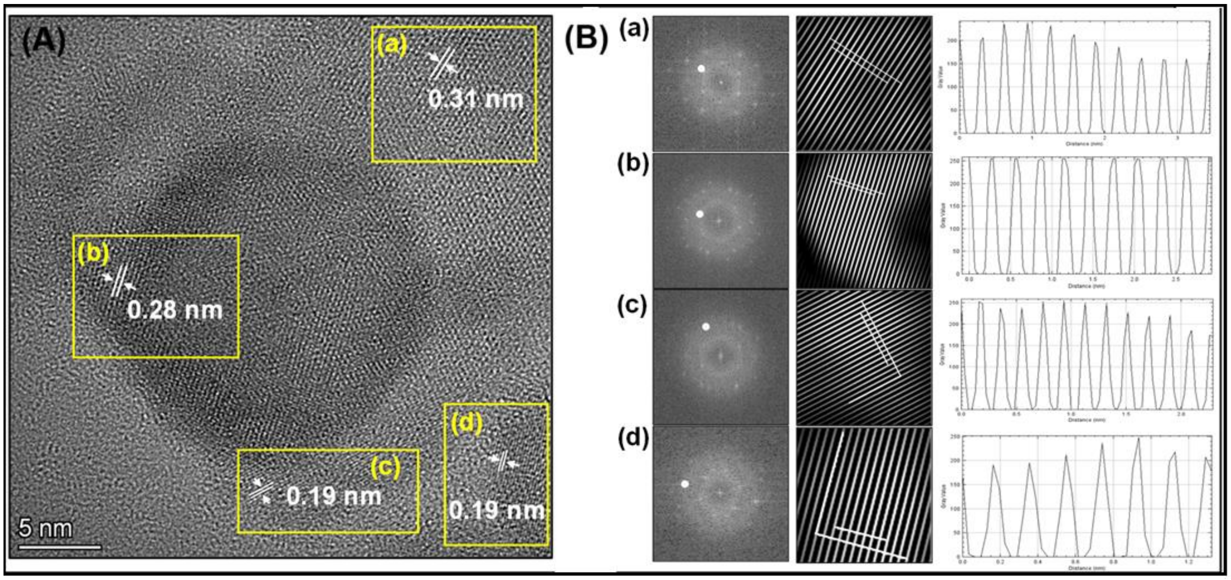

Figure S1. (A) HRTEM image of PEA-CuS NPs showing interplanar spacing (marked at different region) and (B) their respective FFT (first column), IFFT (second column) and profile plot (third column) along marked line in IFFT image.

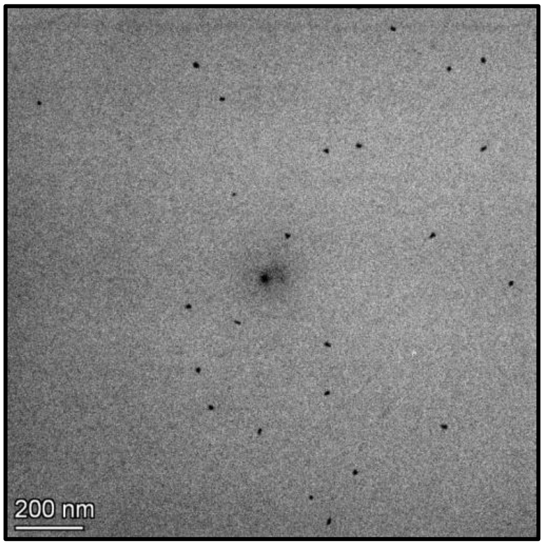

Figure S2. TEM image of PEA-CuS NPs used for obtaining size distribution plot.

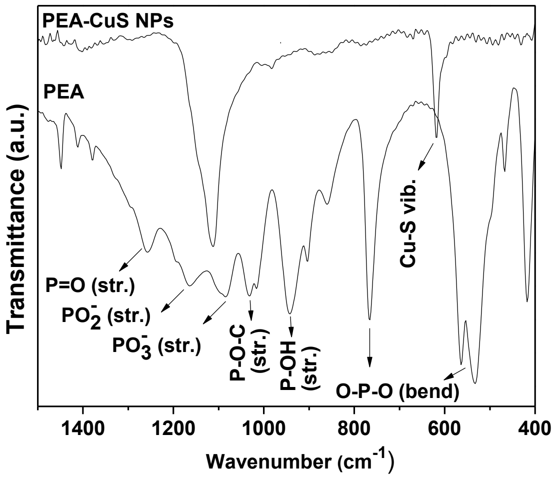

Figure S3. FTIR spectra of PEA and PEA-CuS NPs in the region of 1500-400 cm^{-1} with corresponding band assignments.

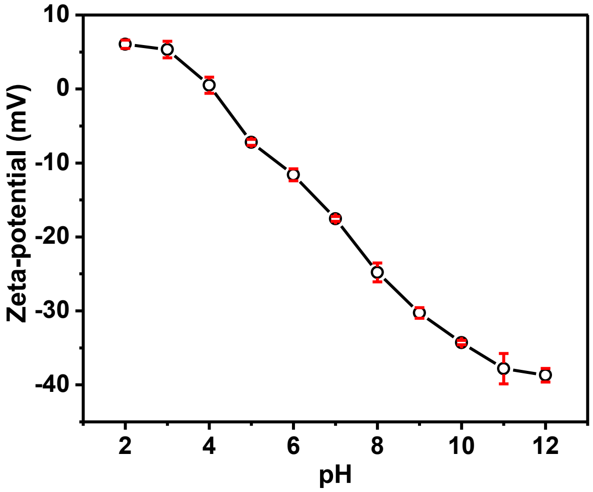

Figure S4. pH dependent zeta-potential of aqueous suspension of PEA-CuS NPs.

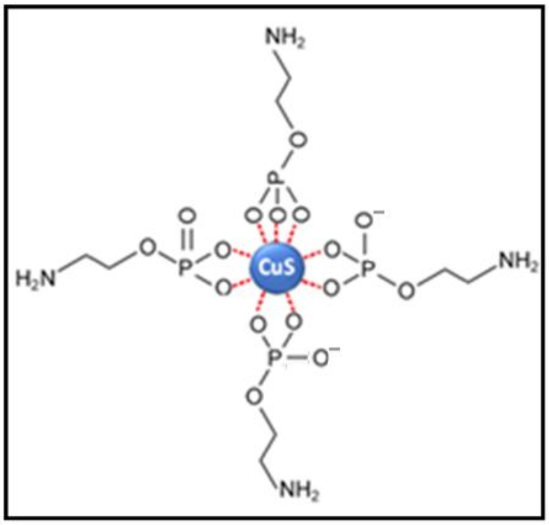

Figure S5. Schematic representation revealing probable Cu-phosphate coordinative coupling of PEA-CuS NPs.

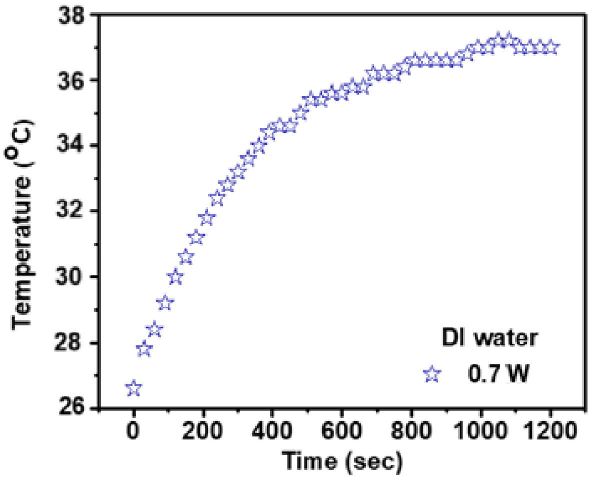

Figure S6. Heating profile of 1ml of DI water under 980 nm (NIR irradiation) laser of power of 0.65 W.

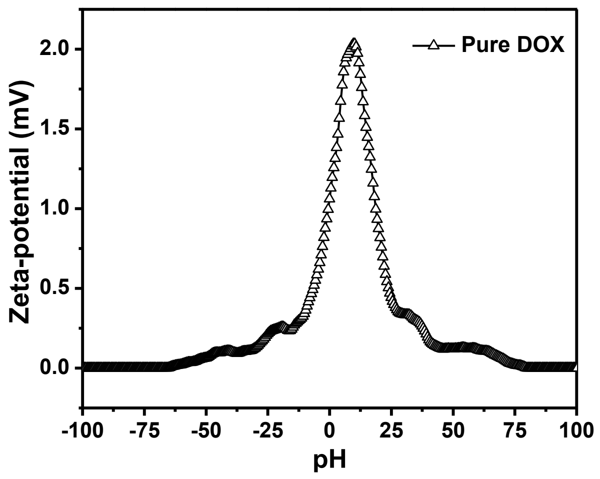

Figure S7. Zeta potential plot of aqueous solution of pure DOX.

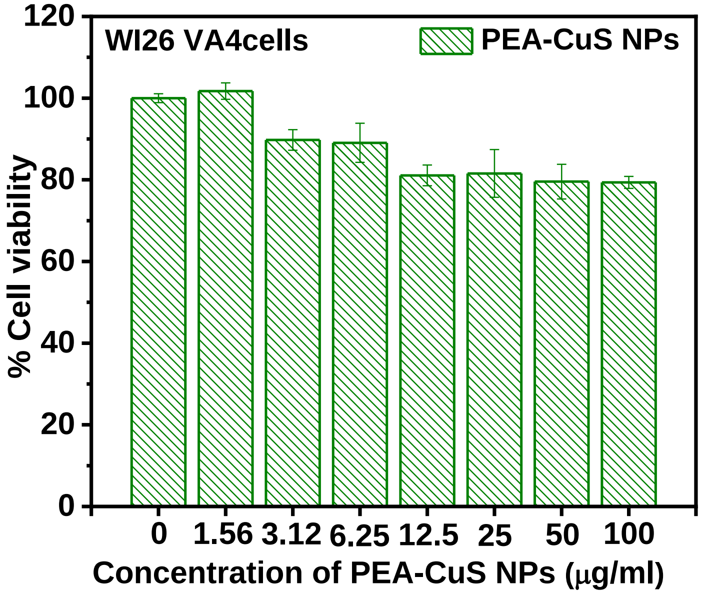

Figure S8. Cell viability of WI26VA4 with treatment of PEA-CuS NPs for 24 h at 37 ˚C and 5% CO_2 under cell culture conditions.

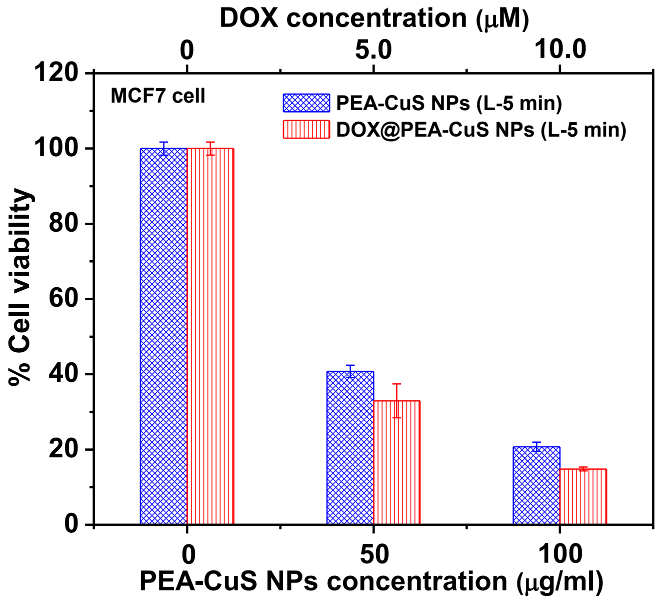

Figure S9. Viability of MCF-7 cells upon incubated with PEA-CuS NPs and DOX@PEA-CuS NPs under irradiation of NIR light (L-5 min).
